# Live Birth in Sex-Reversed XY Mice Lacking the Nuclear Receptor Dax1

**DOI:** 10.1038/s41598-020-58788-9

**Published:** 2020-02-03

**Authors:** Isabel Fernandes-Freitas, Alexandra Milona, Kevin G. Murphy, Waljit S. Dhillo, Bryn M. Owen

**Affiliations:** 1 Section of Endocrinology & Investigative Medicine, Division of Diabetes, Endocrinology, and Metabolism, Department of Metabolism, Digestion, and Reproduction, Imperial College London, London, United Kingdom; 2 MRC London Institute of Medical Sciences (LMS), London, United Kingdom; 3 Institute of Clinical Sciences (ICS), Faculty of Medicine, Imperial College London, London, United Kingdom

**Keywords:** Transcription, Endocrine reproductive disorders

## Abstract

The nuclear hormone receptor Dax1 functions during development as a testes-determining gene. However, the phenotype of male mice lacking Dax1 is strain-dependent due to the background-specific abundance of male-determining *Sry* gene*-*transcripts. We hypothesised that inter-individual variation in *Sry* mRNA-abundance would result in a spectrum of phenotypes even within-strain. We found that while all XY C57BL/6J mice lacking Dax1 presented as phenotypic females, there was a marked inter-individual variability in measures of fertility. Indeed, we report rare occasions where sex-reversed mice had measures of fertility comparable to those in control females. On two occasions, these sex-reversed XY mice were able to give birth to live offspring following mating to stud-males. As such, this work documents within-strain variability in phenotypes of XY mice lacking Dax1, and reports for the first time a complete sex-reversal capable of achieving live birth in these mice.

## Introduction

Dax1 is an orphan member of the nuclear-receptor class of transcription factors^1^. Its gene, *Nr0b1*, is found on the X-Chromosome and encodes a 470 amino acid protein consisting of an N-terminal DNA-binding domain, and a C-terminal region containing a putative ligand-binding domain. Classically, Dax1 represses the transcriptional-activation function of nuclear receptors through protein-protein interactions^2^. However, it has also been reported to activate transcription, and have other functions through its ability to directly bind DNA^3^. In adults, *Dax1* is expressed throughout the hypothalamic-pituitary-gonadal (HPG) axis^4^. It interacts with several steroidogenic receptors including those for estrogen and testosterone^5^. However, the role of Dax1 in the adult HPG axis is largely unknown.

During development, *Dax1* is a testes-determining gene^3,6,7^. While XX Dax1-KO mice are overtly fertile, XY mice lacking Dax1 display a spectrum of sex-reversal phenotypes apparently depending on the animal’s background-strain. For example, XY 129 Sv/J mice lacking exon-2 of *Dax1* have abnormal testicular development resulting in smaller than normal adult testes^8^. By contrast, XY mice lacking *Dax1* in a mixed genetic background, and containing the ‘weak’ *Sry* allele from the *Mus domesticus poschiavinus* Y-Chromosome, display complete sex-reversal^6,7^. Furthermore, ovary development in B6 mice lacking *Dax1* can be prevented by the addition of multiple copies of the male-determining *Sry* allele from the 129-strain^7^. As such, it has been suggested that *Dax1*-deficiency can be bypassed by overexpression of *Sry* gene-transcripts. Importantly, developmental *Sry-*expression is also variable between individuals of the same strain^7^. It is therefore possible that a range of sex-reversal phenotypes may occur even within-strain in XY Dax1-KO mice. However, this has not previously been investigated.

We surveyed reproductive function in chromosomal-XY C57BL/6J mice lacking exon 2 of the *Dax1* gene, henceforth referred to as *Dax1*−/*Y*. At weaning, all of these animals were phenotypically female. However, they displayed a spectrum of reproductive phenotypes and molecular measures of fertility. Many *Dax1*−/*Y* mice were completely infertile. However, a minority of sex-reversed chromosomal-XY *Dax1*−/*Y* mice had measures of fertility comparable to those in control female mice. Indeed, two out of a total of 18 Dax1−/Y mice became pregnant and gave birth to live offspring after mating with stud-males. As such, this work demonstrates intra-strain variability in sex-reversal phenotypes of chromosomal-XY mice lacking *Dax1*. It also reports for the first time a live birth from a sex-reversed chromosomal-male mouse lacking this orphan nuclear receptor.

## Results

We generated C57BL/6J mice lacking exon 2 of the *Dax1* (*Nr0b1*) gene, as described^7^. In accordance with previous findings, all of these *Dax1*−/*Y* mice presented as phenotypic females at weaning.

**Figure 1:**
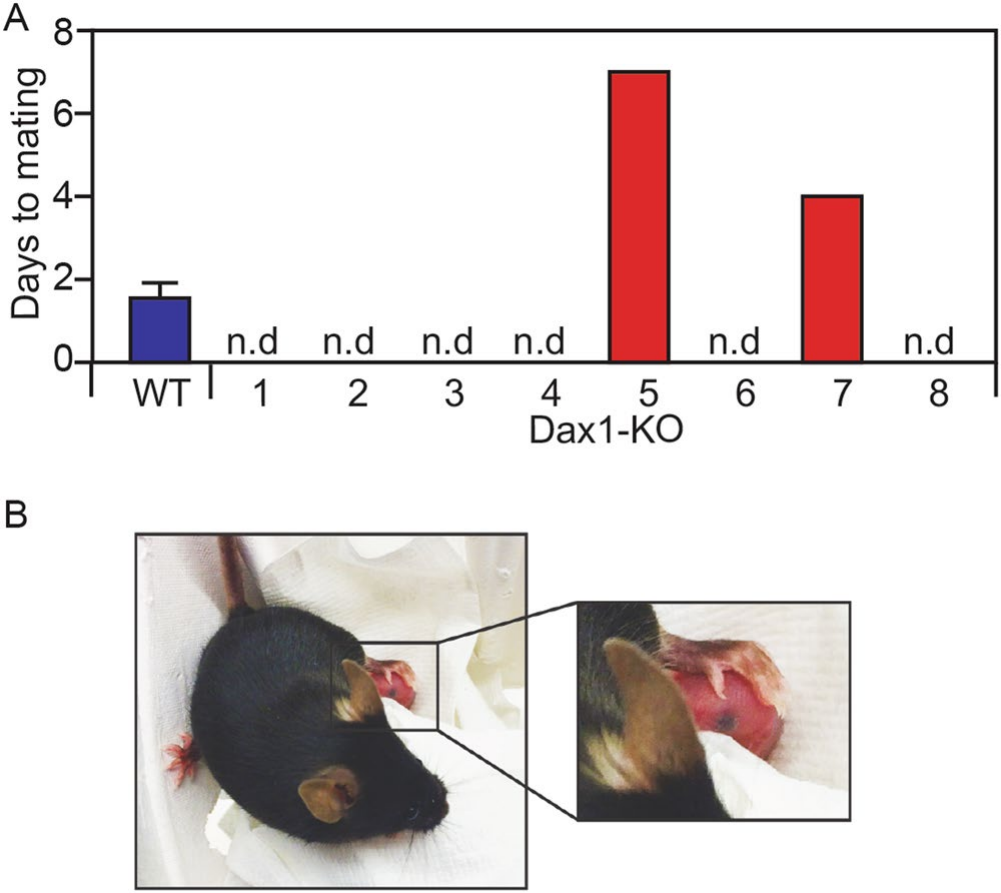
Live-birth in a *Dax1*−/*Y* mouse. (**A**) Mating success of four WT (*Dax1*+/+) females presented as average +/− SEM and eight *Dax1*−/*Y* mice with proven stud-males. The Y axis shows the number of days that a test-mouse was mated with a stud-male before a vaginal plug was detected. (**B**) Photograph of a *Dax1*−/*Y* mouse with single live-offspring on post-natal day 1.

In a preliminary experiment to test fertility, we paired 10 *Dax1*−/*Y* mice with proven *Dax1*+/*Y* stud-males. Approximately two months after pairing, one *Dax1*−/*Y* mouse gave birth to a single offspring which was found dead in the home-cage (data not shown). To confirm this finding, we set up a second fertility experiment involving eight *Dax1*−/*Y* mice and four control (*Dax1*+/+) females. All of the control female mice mated with the stud-males (Fig. 1A). By contrast, only two out of eight *Dax1*−/*Y* mice mated with the stud-males during the 10-day test period (Fig. 1A). One of these animals became pregnant and gave birth to an apparently healthy single offspring (Fig. 1B). For an undetermined reason, the pup did not survive beyond postnatal day two. Therefore, a minority of sex-reversed *Dax1*−/*Y* mice can give birth to live offspring.

**Figure 2:**
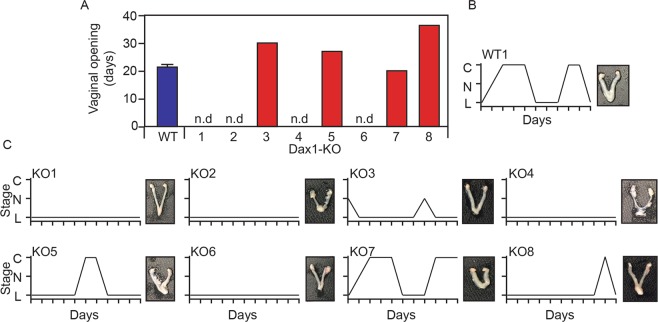
Intra-strain variability in reproductive phenotype of *Dax1*−/*Y* mice. (**A**) Age at first-day of puberty as measured by vaginal opening. WT (*Dax1*+/*+*) females are presented as average +/− SEM of four animals. (**B**) Representative estrous cycle and uterus/ovary morphology in a WT (*Dax1*+/+) female mouse. (**C**) Individual estrous cycles and uterus/ovary morphology in each *Dax1*−/*Y* mouse. C, observation of cornified cells by vaginal cytology. N observation of nucleated cells by vaginal cytology. L observation of leukocytes by vaginal cytology.

The results of our mating-experiments were suggestive of intra-strain variability in the sex-reversal phenotype of *Dax1*−/*Y* mice. In order to investigate the spectrum of reproductive phenotypes in Dax1−/Y mice, we conducted experiments in an additional cohort of four control *Dax1*+/+ female mice and eight sex-reversed *Dax1*−/*Y* mice. We first assessed the timing of puberty by determining vaginal opening^9^. Control females underwent vaginal opening at approximately 21 days of age (Fig. 2A). By contrast, four out of eight *Dax1*−/*Y* mice did not undergo vaginal opening at all during the 40-day test-period (Fig. 2A). Three *Dax1*−/*Y* mice underwent a delayed vaginal opening (between day-26 and day-35), and one *Dax1*−/*Y* mouse underwent normal vaginal opening on day-20 (Fig. 2A).

We next assessed the estrous cyclicity by vaginal cytology. As expected, control female mice underwent a 4–6 day cycle and entered the post-ovulatory estrous-phase twice during the 11-day testing period (Fig. 2B). By contrast, over half (five out of eight) of the *Dax1*−/*Y* mice did not enter the ovulatory phase of the cycle at all during the testing-period (Fig. 2C). Two *Dax1*−/*Y* mice did enter the estrous-phase but did not cycle normally. One *Dax1*−/*Y* mouse cycled normally and entered the estrous-phase twice during the test-period. There was a clear connection between the *Dax1*−/*Y* individuals that underwent vaginal opening (Fig. 2A) and those that had evidence of estrous cyclicity (Fig. 2C). In addition, the gross morphology of the uterus and ovaries reflected the vaginal cytology data. Those animals displaying evidence of estrous cyclicity had larger ovaries and uteri than those that did not (Fig. 2C). Together, these data show clear intra-strain variability in the reproductive phenotype of C5BL/6J *Dax1*−/*Y* mice. A minority of sex-reversed *Dax1*−/*Y* mice are comparable to control females with regard to their time of puberty onset and estrous cyclicity.

**Figure 3:**
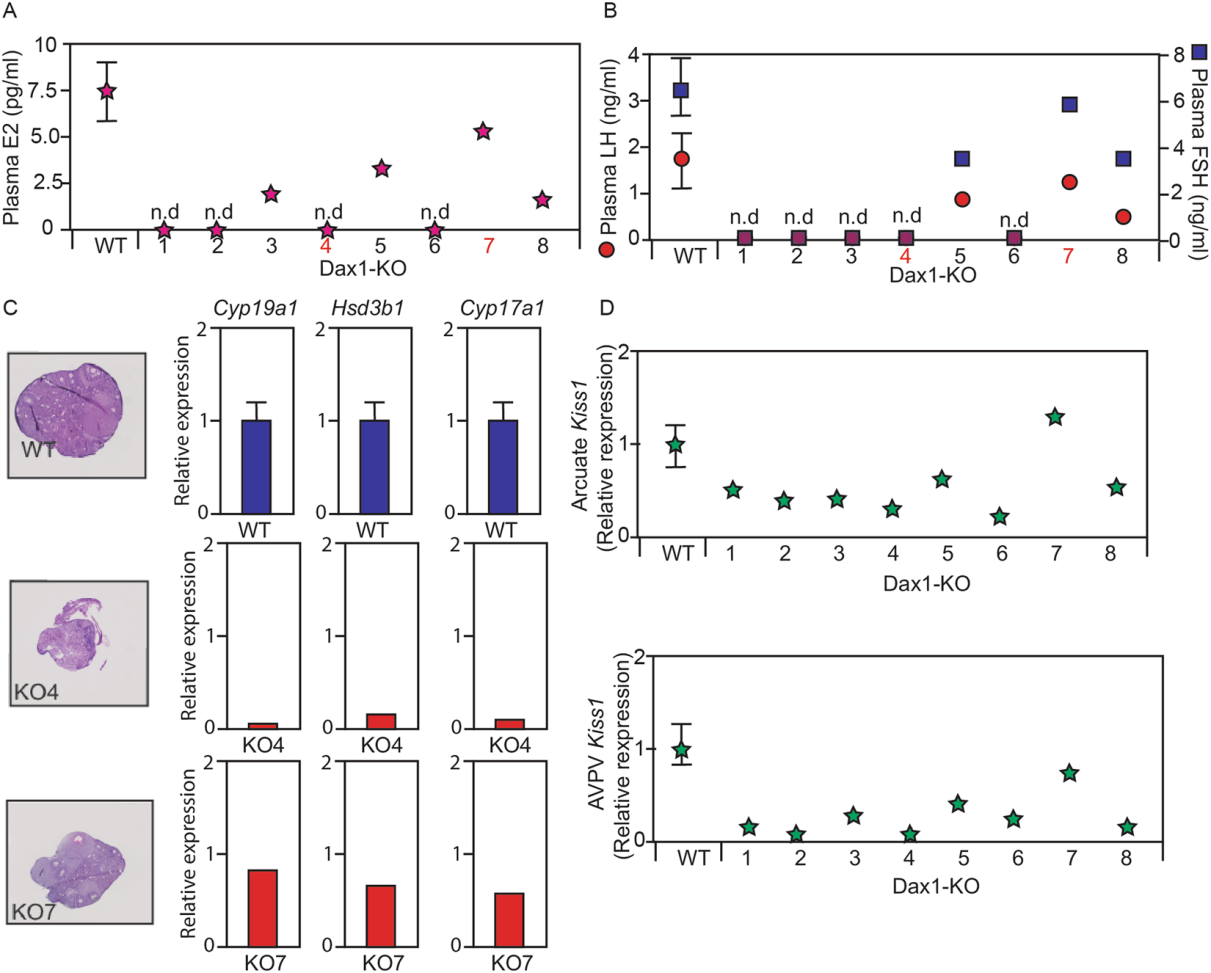
*Dax1*−/*Y* mice respond to ovarian estrogen production. (**A**) plasma estradiol levels. (**B**) Plasma LH and FSH levels in four control females, presented as average +/−  SEM, and eight individual *Dax1*−/*Y mice*. (**C**) Representative ovarian histology and ovarian steroidogenic gene expression in four WT (*Dax1*+/+) female mice (gene expression presented as average +/− SEM), in an acyclic *Dax1*−/*Y* mouse (KO4), and in a cyclic *Dax1*−/*Y* mouse (KO7). (**D**) *Kiss1* gene-expression in the arcuate and AVPV hypothalamus in four control females, presented as average +/− SEM, and eight individual *Dax1*−/*Y mice*.

In order to gain additional insight in to our observations on sex-reversal phenotypes, we conducted post-mortem analysis of plasma hormone levels, ovaries, and hypothalamic neuropeptide gene-expression. Plasma estrogen (E2), luteinizing hormone (LH), and follicle-stimulating hormone (FSH) were all significantly lower (p < 0.05) in *Dax1*−/*Y* mice than in control females. Indeed, these hormones were undetectable in at least half of the *Dax1*−/*Y* mice (Fig. 3A,B). However, E2, LH, and FSH were detectible in three *Dax1*−/*Y* mice and their levels were in a range that could be considered ‘normal’ (Fig. 3A,B). Examples of ovarian histology and steroidogenic gene-expression from a control female, an acyclic *Dax1*−/*Y* mouse, and a cyclic *Dax1*−/*Y* mouse are provided in Fig. 3C. The acyclic *Dax1*−/*Y* mouse had underdeveloped ovaries with few mature follicles or corpora lutea (Fig. 3C). It also had dramatically reduced expression of key steroidogenic genes (*Cyp19a1*, *Hsd3b1*, and *Cyp17a1*) (Fig. 3C). By contrast, the cyclic *Dax1*−/*Y* mouse had overtly normal ovaries and expression of *Cyp19a1*, *Hsd3b1*, and *Cyp17a1* was comparable to controls (Fig. 3C).

Kisspeptin (*Kiss1*) is a neuropeptide that controls fertility by signalling to gonadotropin-releasing hormone (GnRH) neurons. In female mice, plasma gonadotropins (FSH and LH) are controlled by *kiss1*-neurons in the arcuate nucleus (Arc) and anteroventral periventricular nucleus (AVPV) of the hypothalamus respectively. Hypothalamic *Kiss1* expression was broadly in concordance with plasma gonadotropins in *Dax1*−/*Y* mice. While the majority of these animals had lower *Kiss1* expression than control mice, some had broadly normal *Kiss1* gene expression in both the Arc and AVPV (Fig. 3D). Perturbations in *Kiss1*-expression in *Dax1*−/*Y* mice were more dramatic in AVPV than in the Arc (Fig. 3D). Furthermore, there is apparent concordance between plasma E2 levels, plasma gonadotropins, and hypothalamic *Kiss1*-expression in individual *Dax1*−/*Y* mice (Fig. 3A,B,D). This suggests that the hypothalamus of *Dax1*−/*Y* mice is able to respond normally to ovarian sex steroids.

Taken together, these data provide the first evidence of dramatic intra-strain variation in markers of fertility in sex-reversed chromosomal-XY mice lacking the nuclear receptor Dax1. They also provide additional evidence that the primary defect in these animals is ovarian. Finally, we show that a minority of these sex-reversed chromosomal-XY mice can undergo puberty, maintain pregnancy, and give birth to live offspring.

## Discussion

Our results confirm that the C57BL/6J background is highly sensitive to male-to-female sex-reversal^10^. *Dax1*−/*Y* mice on this strain present at weaning as phenotypic females. However, there is marked inter-individual variation in reproductive phenotypes. Most *Dax1*−/*Y* mice are infertile but a minority can give birth to live offspring.

It has been suggested that differences in reproductive phenotype amongst different strains of *Dax1*−/*Y* mice is due to the relative ‘strength’ (amount of transcript) of the male-determining *Sry* allele^6,7^. Indeed, overexpression of *Sry* in a strain susceptible to sex-reversal prevents ovary formation in *Dax1*−/*Y* mice^7^. However, *Sry* mRNA expression has been shown to be variable even within-strain during the early events of testicular development^7^, and therefore provides a logical mechanistic explanation for the intra-strain variation in phenotypes observed here. Unfortunately, it is not currently possible to correlate early developmental expression of Sry in the testes to the degree of sex-reversal observed in adult *Dax1*−/*Y* mice. In addition, variability in sex-reversal has been observed on a number of other occasions in other models and possibly involves stochastic expression of pro-ovary genes^11,12^.

We found that most *Dax1*−/*Y* mice present with apparent hypothalamic hypogonadism. They have low plasma gonadotropins, and low estradiol levels. However, there was good concordance between plasma E2, hypothalamic *Kiss1*-expression, and circulating gonadotropins amongst individual *Dax1*−/*Y* mice (Fig. 3). This suggests that hypothalamic hypogonadism in these animals is secondary to ovarian dysfunction. In other words, individual *Dax1*−/*Y* mice that are able to produce ovarian E2 are able to respond to it appropriately. Furthermore, the fact that *Kiss1*-expression in the AVPV is more dramatically affected than *Kiss1*-expression in the Arc of *Dax1*−/*Y* mice is consistent with the hypothesis that the AVPV is a female-predominant nucleus which develops under the control of estrogen.

Although two out of a total of 18 *Dax1*−/*Y* mice gave birth, they each produced only one offspring, and neither animal survived beyond post-natal day two. As such, although a minority of *Dax1*−/*Y* mice have markers of fertility that are broadly in line with control females, they are still dramatically sub-fertile. The reasons for this are currently unclear but are probably due to a combination of factors that are likely a result of abnormal ovarian development. However, the mechanistic presence of a Y chromosome has also been suggested to inhibit meiosis in females^13–15^. Indeed, while we did detect DNA from the Y chromosome in an ovary from a sex reversed *Dax1*−/*Y* mouse that gave birth, this does not exclude the possibility that some ovarian cells in this animal may have lost the Y chromosome (XO).

This work demonstrates that a minority of *Dax1*−/*Y* mice can produce live offspring without any assisted fertility treatment. It adds to the small number of reports of live-birth from sex-reversed XY mice (for example^16–19^). There are reports of fertility in mutant XY sex-reversed wood lemmings (*Myopus shisticolor*)^20^, and XY-female horses^21^. In addition, we are aware of one remarkable report of a fertile woman with normal ovaries and a predominantly XY karyotype^22^. As such, our observations in *Dax1*−/*Y* mice support the notion that XY sex-reversal is not formally incompatible with reproduction. However, our data also suggest that the inter-individual milieu of mechanical and development complications caused by the presence of the Y-chromosome makes successful reproduction highly unlikely.

## Methods

### Mice

All procedures were conducted in accordance with the U.K Animals (Scientific Procedures) Act 1986, with ethical approval from the Institutional Animal Care and Welfare Committee at Imperial College London. Mice were housed under standard conditions under a 12 h light-dark cycle and with free access to water and soy-free diet. C57BL/6J mice with deletion of *Dax1* (*Nr0b2*) were generated essentially as described previously and had undergone between 8 and 12 back-crosses on the C57BL/6J line^7^.

### Mating experiments

Individual female *Dax1*+/+ mice, and *Dax1 −*/*Y* mice were housed with a proven stud-male. Mating-success was determined by the presence of a vaginal plug in the morning during a ten-day mating experiment.

### Puberty onset and estrus cyclicity

The day of vaginal opening (first day of puberty) and the stage of the estrous cycle were determined by cytology using standard methods.

### Hormone analysis

Plasma estradiol was determined by ELISA (R&D Systems). Plasma LH and FSH were determined by the University of Virginia Center for Research in Reproduction Ligand Assay and Analysis Core.

### Tissue collection and gene expression analysis

Tissues were collected in diestrus and either snap-frozen in liquid nitrogen or placed in buffered formalin for later histological analysis. Ovaries were dehydrated in ethanol and mounted in paraffin blocks. Sections were mounted on glass slides and stained using haematoxylin and eosin by standard methods. Ovarian and hypothalamic gene expression were determined following RNA extraction (Ambion PureLink), cDNA synthesis (Invitrogen), SYBR-Green qPCR (Sigma-Aldrich) and expressed as relative expression after correcting for internal control. *Cyclophilin* was used as an internal control. Primer-sequences for gene expression are available on request.

### Statistical analysis

Statistical analysis were performed where appropriate by students t-test, with a p < 0.05 being considered statistically significant.

## Data Availability

All data will be made available upon reasonable request.

## References

[1] Mangelsdorf DJ (1995). The nuclear receptor superfamily: the second decade. Cell.

[2] Crawford PA, Dorn C, Sadovsky Y, Milbrandt J (1998). Nuclear receptor DAX-1 recruits nuclear receptor corepressor N-CoR to steroidogenic factor 1. Mol Cell Biol.

[3] Ludbrook LM, Harley VR (2004). Sex determination: a ‘window’ of DAX1 activity. Trends Endocrinol Metab.

[4] Ikeda Y (2001). Comparative localization of Dax-1 and Ad4BP/SF-1 during development of the hypothalamic-pituitary-gonadal axis suggests their closely related and distinct functions. Dev Dyn.

[5] Iyer AK, McCabe ER (2004). Molecular mechanisms of DAX1 action. Mol Genet Metab.

[6] Meeks JJ, Weiss J, Jameson JL (2003). Dax1 is required for testis determination. Nat Genet.

[7] Bouma GJ (2005). Gonadal sex reversal in mutant Dax1 XY mice: a failure to upregulate Sox9 in pre-Sertoli cells. Development.

[8] Yu RN, Ito M, Saunders TL, Camper SA, Jameson JL (1998). Role of Ahch in gonadal development and gametogenesis. Nat Genet.

[9] Vazquez MJ (2018). SIRT1 mediates obesity- and nutrient-dependent perturbation of pubertal timing by epigenetically controlling Kiss1 expression. Nat Commun.

[10] Munger SC, Natarajan A, Looger LL, Ohler U, Capel B (2013). Fine time course expression analysis identifies cascades of activation and repression and maps a putative regulator of mammalian sex determination. PLoS Genet.

[11] Harris A (2018). ZNRF3 functions in mammalian sex determination by inhibiting canonical WNT signaling. Proc Natl Acad Sci USA.

[12] Larney C, Bailey TL, Koopman P (2014). Switching on sex: transcriptional regulation of the testis-determining gene Sry. Development.

[13] Obata Y, Villemure M, Kono T, Taketo T (2008). Transmission of Y chromosomes from XY female mice was made possible by the replacement of cytoplasm during oocyte maturation. Proc Natl Acad Sci USA.

[14] Taketo T (2015). The role of sex chromosomes in mammalian germ cell differentiation: can the germ cells carrying X and Y chromosomes differentiate into fertile oocytes?. Asian J Androl.

[15] Vernet N (2014). The expression of Y-linked Zfy2 in XY mouse oocytes leads to frequent meiosis 2 defects, a high incidence of subsequent early cleavage stage arrest and infertility. Development.

[16] Eicher EM, Washburn LL, Whitney JB, Morrow KE (1982). Mus poschiavinus Y chromosome in the C57BL/6J murine genome causes sex reversal. Science.

[17] Lovell-Badge R, Robertson EXY (1990). female mice resulting from a heritable mutation in the primary testis-determining gene, Tdy. Development.

[18] Kuno J (2015). Generation of fertile and fecund F0 XY female mice from XY ES cells. Transgenic Res.

[19] Kuroki S (2013). Epigenetic regulation of mouse sex determination by the histone demethylase Jmjd1a. Science.

[20] Liu WS, Eriksson L, Fredga K (1998). XY sex reversal in the wood lemming is associated with deletion of Xp21-23 as revealed by chromosome microdissection and fluorescence *in situ* hybridization. Chromosome Res.

[21] Sharp AJ, Wachtel SS, Benirschke K (1980). H-Y antigen in a fertile XY female horse. J Reprod Fertil.

[22] Dumic M (2008). Report of fertility in woman with predominantly 46,XY karyotype in family with multiple disorders of sexual development: review of prismatic case. Mt Sinai J Med.

